# Use of Handheld Bedside Ultrasound to Confirm Successful Reduction of an Anterior Shoulder Dislocation

**DOI:** 10.7759/cureus.52089

**Published:** 2024-01-11

**Authors:** Robin Lahr, Frank Wheeler, James Espinosa, Alan Lucerna, Henry Schuitema

**Affiliations:** 1 Emergency Medicine, Jefferson Health, Stratford, USA

**Keywords:** handheld ultrasound in shoulder dislocations, handheld ultrasound, bedside ultrasound, shoulder dislocation, anterior shoulder dislocation

## Abstract

We present the case of a 30-year-old male with anterior shoulder dislocation in which a bedside handheld ultrasound was used after sedation and a reduction procedure to confirm successful reduction. X-ray imaging as well as bedside ultrasound was performed before and after the reduction. The bedside handheld ultrasound demonstrated findings comparable to the X-ray results. X-ray imaging is used as a standard not only in the diagnosis of a dislocated shoulder but also to ensure successful reduction and to assess for any procedure-related fractures. An advantage of immediate bedside ultrasound is that immediate recognition by ultrasound of an unsuccessful reduction can allow the reduction process to continue while the patient is sedated, thus avoiding additional independent sedation procedures. The utilization of bedside ultrasonography in this manner may allow more expeditious and safer care for patients with shoulder dislocations.

## Introduction

The glenohumeral joint has the largest range of motion of any joint, and it is no surprise that it is the most commonly dislocated joint as well [1]. The glenohumeral joint dislocates at an incidence of 11-24 per 100,000 person-years [2]. Multiple studies have demonstrated the accuracy of point-of-care ultrasound (POCUS) in the diagnosis of shoulder dislocations, with sensitivities and specificities approaching 100% in multiple reports [3-7]. In this novel case study, we demonstrate the use of a handheld bedside ultrasound to confirm shoulder reduction prior to X-ray imaging being completed.

## Case presentation

A 30-year-old male with a past medical history of multiple left shoulder dislocations presented for shoulder pain following a motorcycle accident. The patient reported that he was driving his motorcycle when it slid, and he lost control. The patient located the pain in his left shoulder. He stated that he had difficulty moving his shoulder following the injury. He denied other injuries. His vital signs on arrival were as follows: BP 145/97 mmHg, HR 96 bpm, temperature 98.5° F, oxygen saturation 99%, and respiration rate 16 breaths per minute.

**Figure 1:**
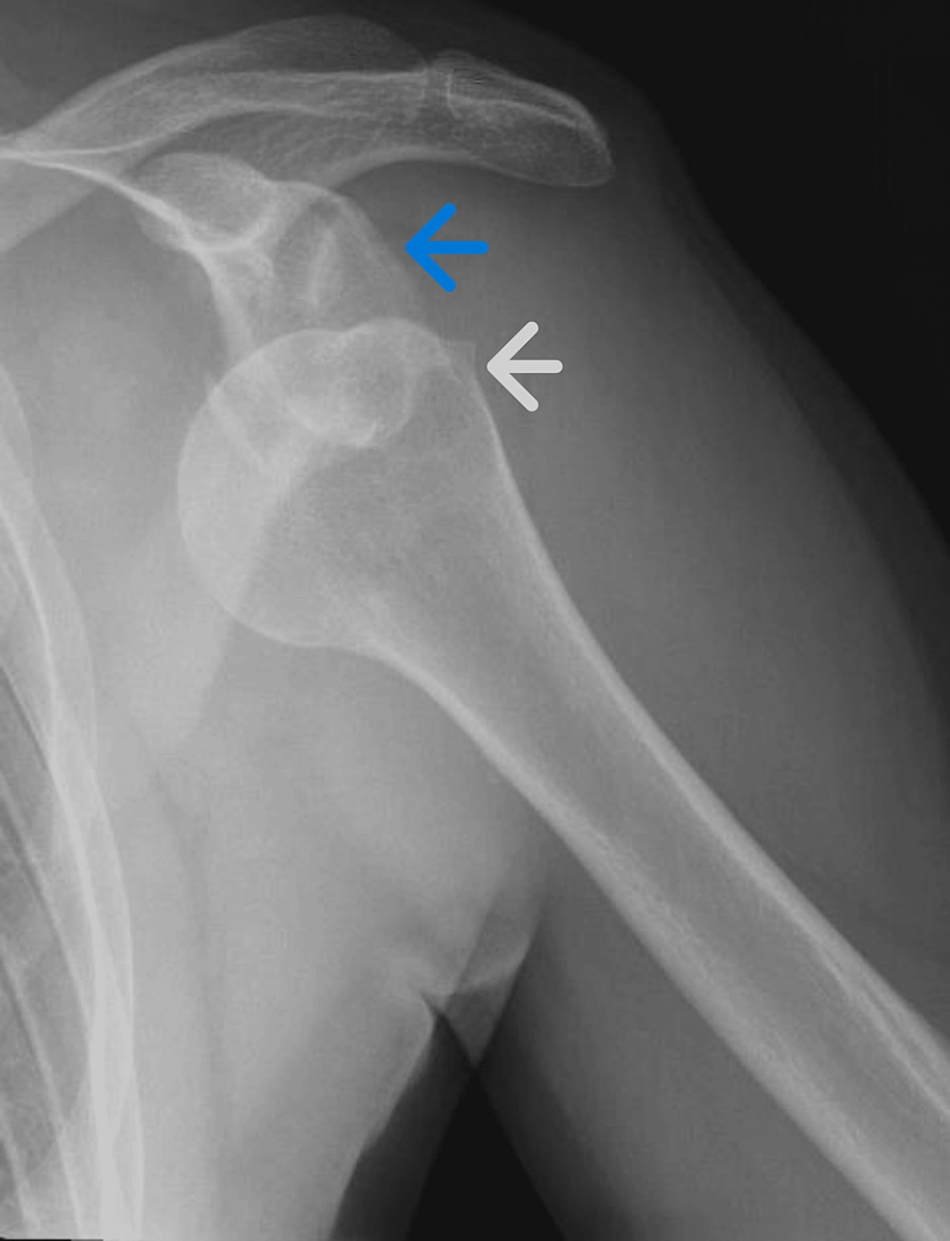
X-ray prior to reduction showing anterior shoulder dislocation Glenoid fossa (blue arrow); humeral head (white arrow)

Upon examination, the patient held the affected left arm in internal rotation with flexion at the elbow. The affected extremity was neurovascularly intact. An X-ray demonstrated anterior shoulder dislocation (Figure 1).

**Figure 2:**
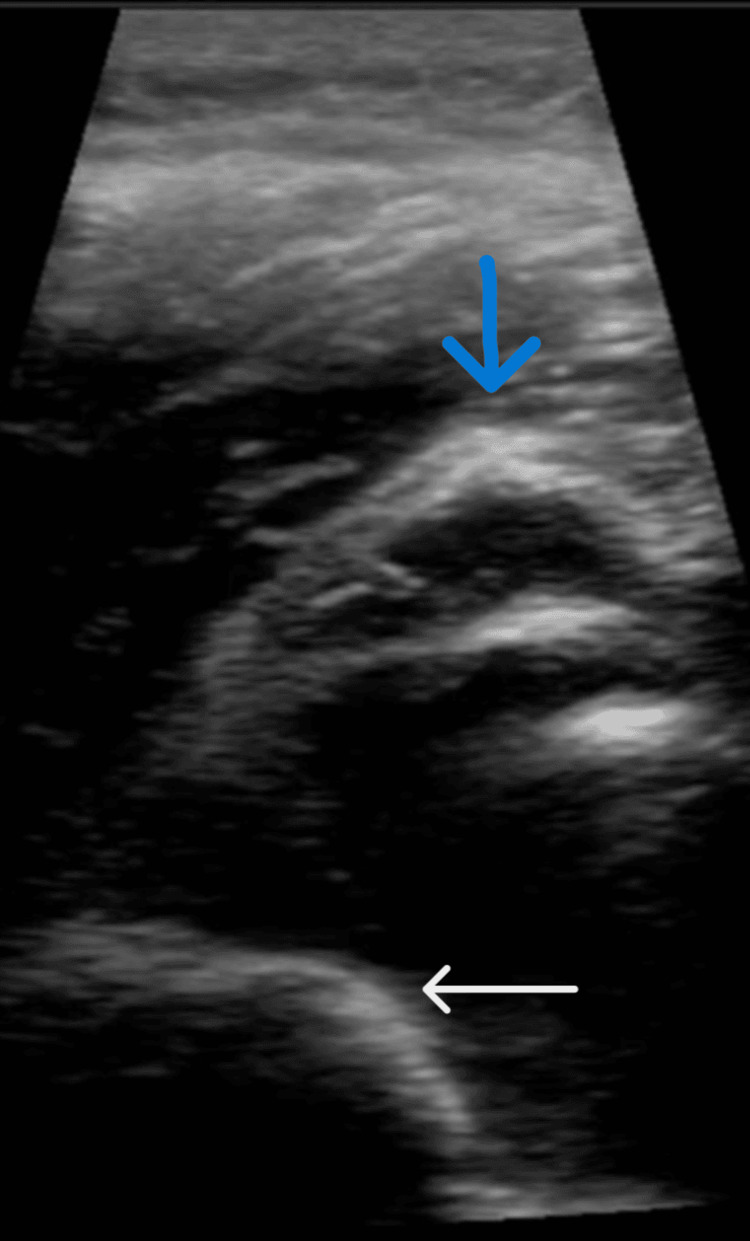
2: Bedside portable ultrasound demonstrating the humeral head (white arrow) anterior to the scapular spine (blue arrow), indicating anterior shoulder dislocation

Bedside handheld ultrasound was then utilized with the probe placed on the patient’s posterior shoulder joint along the scapular spine. The probe was adjusted until the glenoid fossa and humeral head could be visualized. The humeral head was anterior to the glenoid fossa, indicating anterior shoulder dislocation (Figure 2).

No fracture was visualized on either ultrasound or X-ray.

**Figure 3:**
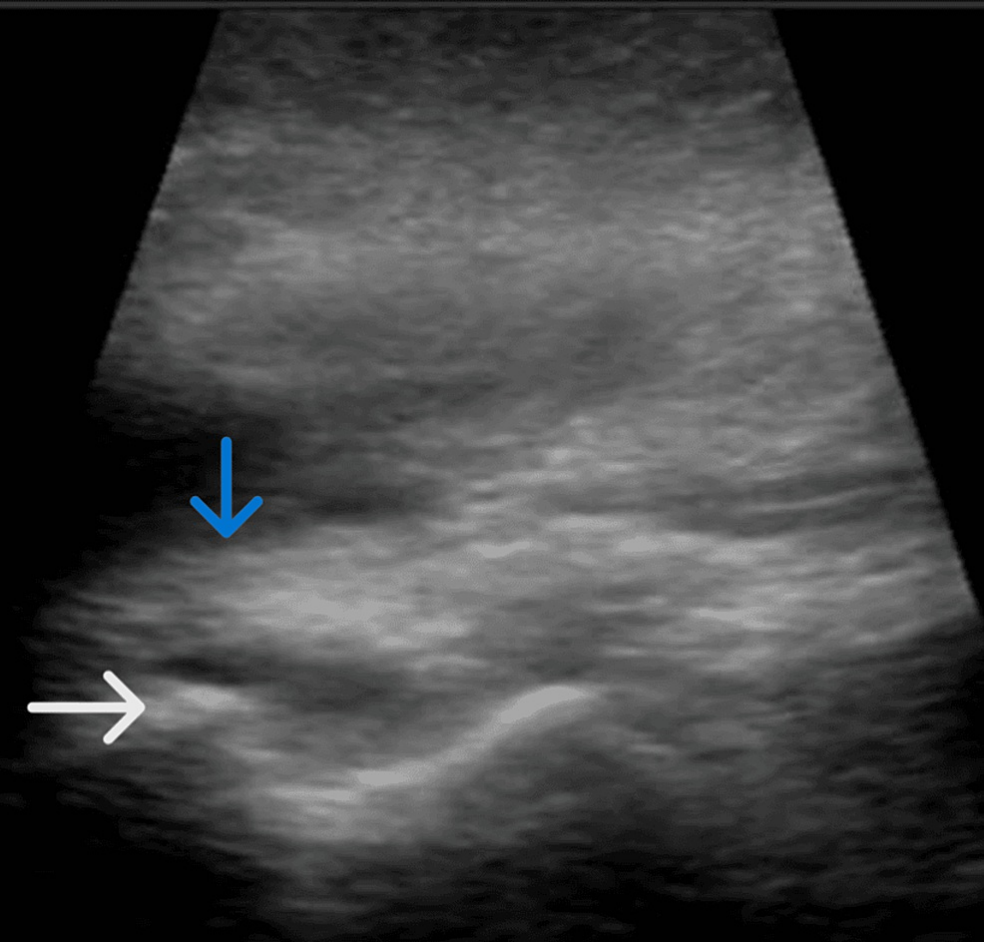
Bedside portable ultrasound demonstrating the humeral head (white arrow) in normal alignment with the scapular spine (blue arrow), indicating the successful reduction of the anterior dislocation

Consent was obtained prior to sedation and reduction. The patient was sedated and the affected shoulder was reduced. Prior to post-reduction X-ray imaging, a bedside portable ultrasound was performed, which demonstrated the successful reduction of the dislocated shoulder (Figure 3).

**Figure 4:**
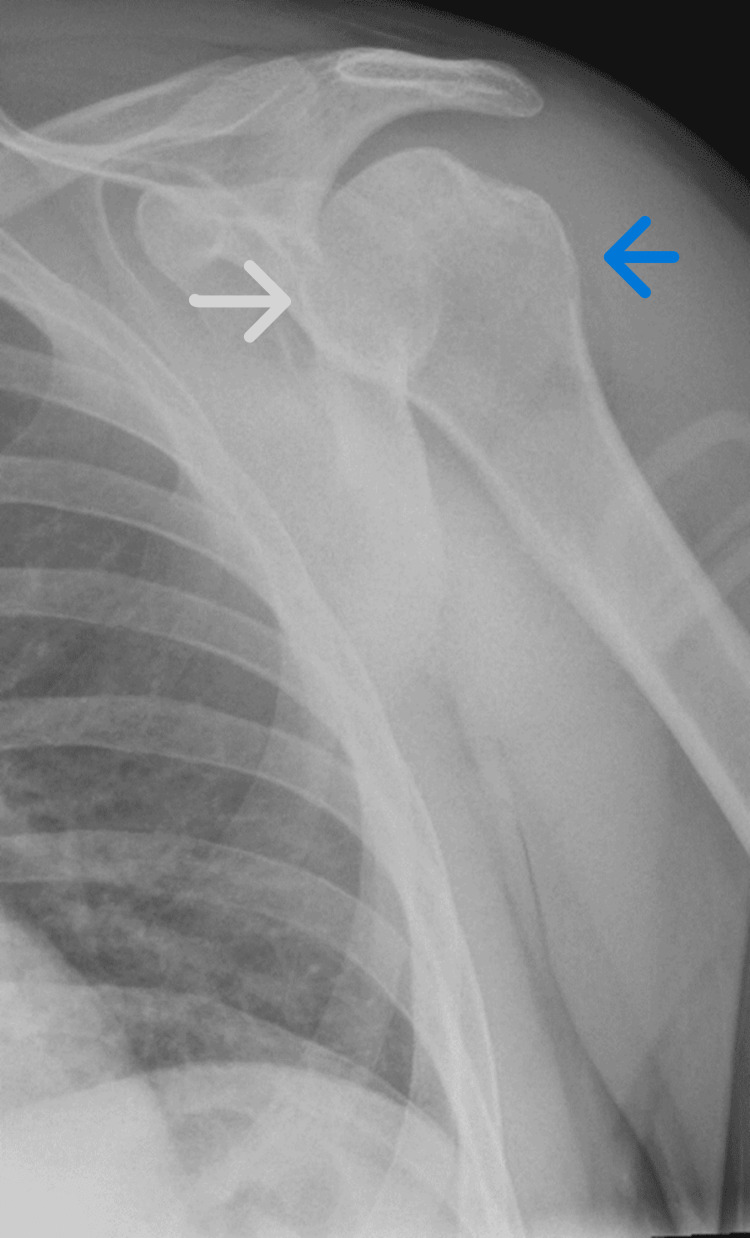
X-ray demonstrating successful reduction of the anterior dislocation Normal relationship now seen between the humeral head (blue arrow) and the glenoid fossa (white arrow).

X-ray imaging subsequently demonstrated congruent findings to the bedside portable ultrasound (Figure 4).

The patient was monitored and discharged with instructions to follow up with orthopedics.

## Discussion

The glenohumeral joint has the largest range of motion of any joint and is the most commonly dislocated joint [1]. The glenohumeral joint dislocates at an incidence of 11-24 per 100,000 person-years [2]. As demonstrated in the case presented, point-of-care ultrasound (POCUS) can be performed not only to diagnose shoulder dislocations but also to confirm a successful reduction of a dislocated shoulder.

Multiple studies have demonstrated the accuracy of POCUS in the diagnosis of shoulder dislocations with sensitivities and specificities approaching 100% in multiple reports [3-7]. Not only has POCUS been shown to be accurate in diagnosing shoulder dislocations, but even novice sonographers with limited ultrasound training have been shown to be able to utilize this diagnostic modality effectively [8]. Seyedhosseini et al. demonstrated that POCUS could also be utilized to demonstrate the successful reduction of shoulder dislocation with a specificity of 98.7 and a negative predictive value of 100% [9]. Although the literature is sparse in regard to the use of handheld ultrasound devices to confirm successful reduction attempts, a small number of case reports demonstrate that handheld ultrasound can be used to verify the reduction of a shoulder dislocation [10-11].

As a standard, X-ray imaging is used not only in the diagnosis of a dislocated shoulder but also to ensure a successful reduction and to assess for any procedure-related fractures. However, even with a portable X-ray team, it takes time to perform X-ray imaging. Repeat sedation attempts may be avoided if ultrasound demonstrates that successful reduction has not yet been achieved. The process of ending further sedation can proceed pending x-ray confirmation if the bedside ultrasound indicates successful reduction This case illustrates the usefulness of bedside portable ultrasound in confirming the proper reduction of a dislocated shoulder. The ultrasound assessment was then confirmed by conventional X-ray. Timely confirmation of an unsuccessful reduction can allow the reduction work to continue, while the patient is sedated, thus avoiding additional sedation attempts.

## Conclusions

This paper presents the case of a shoulder dislocation in which handheld bedside ultrasound was utilized to accurately confirm successful reduction. Although further studies are needed, utilizing bedside ultrasonography in this manner may allow us to provide more expeditious and safer care to patients with shoulder dislocations.
